# Treatment of Severe Tumoral Calcinosis with Teriparatide in a Dialysis Patient after Total Parathyroidectomy

**DOI:** 10.1155/2021/6695906

**Published:** 2021-01-06

**Authors:** Ho-Kwan Sin, Ping-Nam Wong, Kin-Yee Lo, Man-Wai Lo, Shuk-Fan Chan, Kwok-Chi Lo, Yuk-Yi Wong, Lo-Yi Ho, Wing-Tung Kwok, Kai-Chun Chan, Andrew Kui-Man Wong, Siu-Ka Mak

**Affiliations:** Department of Medicine and Geriatrics, Kwong Wah Hospital, 25 Waterloo Road, Kowloon, Hong Kong

## Abstract

Tumoral calcinosis is a rare but debilitating condition that can affect dialysis patients. Optimal management is largely unknown. We report the clinical course, treatment, and outcome of a peritoneal dialysis (PD) patient who developed tumoral calcinosis refractory to conventional treatment but improved with teriparatide therapy. A 26-year-old lady on PD for 2 years presented to us with tumoral calcinosis involving bilateral hands. Response to surgical excision, parathyroidectomy, and conversion to hemodialysis failed to result in sustained remission, and tumoral calcinosis progressed. After total parathyroidectomy, the patient had transient but partial remission in which her calcinosis deposits remained but were asymptomatic without pain or clinical signs of inflammation. However, she later experienced a relapse with involvement of the left elbow, right shoulder, right hip, and right thigh. Tumoral calcinosis remained uncontrolled resulting in debilitation, likely attributable to poor calcium and phosphate control because of adynamic bone disease after parathyroidectomy despite treatment of superimposed tuberculosis and therapy with sodium thiosulphate and pamidronic acid. Clinical improvement was however evident after the use of teriparatide. Asymptomatic hypocalcemia occurred after teriparatide therapy but resolved after 2 months. In conclusion, teriparatide appears to be useful for treating tumoral calcinosis in the presence of adynamic bone disease. Hypocalcemia can occur in the initial months of therapy.

## 1. Introduction

Tumoral calcinosis is a rare but debilitating condition that can affect dialysis patients. It is characterized by the deposition of calcium phosphate crystals in the soft tissue and periarticular areas leading to complications such as rupture, infection, and systemic inflammation [1–11]. Treatment is guided by limited evidence but involves restoration of calcium and phosphate balance by an assortment of measures that include intensification of dialysis [2–5, 10], cessation of calcium-based phosphate binders and vitamin D analogues [4, 10], use of non-calcium phosphate binders [1–5, 10], low dialysate calcium [3, 4, 10], use of cinacalcet [6], and parathyroidectomy [6, 11]. Peritoneal dialysis (PD) patients may benefit from a switch to hemodialysis [2]. The use of sodium thiosulphate (STS) and bisphosphonates has been reported [7, 9].

## 2. Case Report

**Figure 1:**
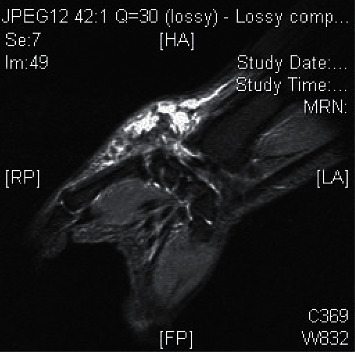
Magnetic resonance imaging (T2-weighted) of the left wrist.

**Figure 2:**
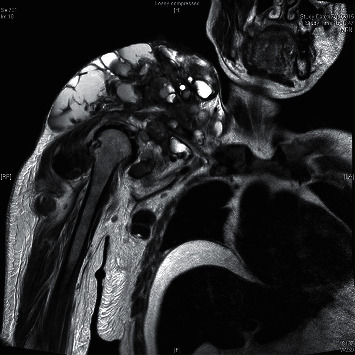
Magnetic resonance imaging (T2-weighted) of the right shoulder.

**Figure 3:**
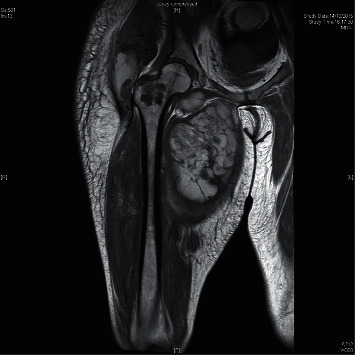
Magnetic resonance imaging (T2-weighted) of the right hip.

**Figure 4:**
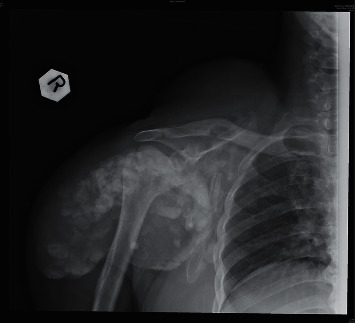
Radiograph of the right shoulder before teriparatide therapy.

**Figure 5:**
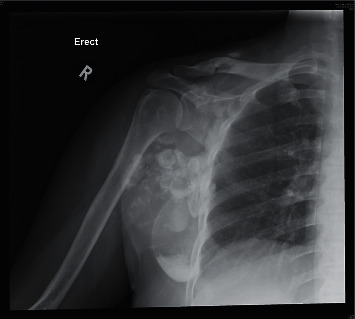
Radiograph of the right shoulder 1 year after teriparatide therapy.

**Figure 6:**
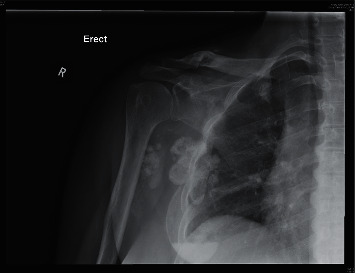
Radiograph of the right shoulder 2 years after teriparatide therapy.

A 26-year-old Chinese lady with end-stage renal failure (ESRD) of unknown cause presented to us in February 2009 with multiple subcutaneous nodules involving the left wrist, left little finger, and right thumb. She had been on continuous ambulatory peritoneal dialysis (CAPD) for 2 years. Her medical history was significant for gout and chronic hepatitis B infection. She had significant hyperphosphatemia and was taking oral calcium carbonate 2 g twice daily as a phosphate binder (Table 1). Radiographs showed calcified soft tissue deposits in periarticular areas of the hands. Magnetic resonance imaging showed T2-inhomogeneous hyperintense calcified deposits around the left wrist joint (Figure 1). The nodules were excised, and histological examination revealed amorphous calcified material deposited in the fibroadipose tissue with surrounding granulomata consistent with tumoral calcinosis. Lanthanum 500 mg thrice daily was given in place of calcium carbonate to lower phosphate, and the CAPD regime was intensified, but tumoral calcinosis persisted over the ensuing months requiring further surgical excision. In view of uncontrolled tumoral calcinosis in the face of worsening secondary hyperparathyroidism (SHPT) (Table 1), she underwent total parathyroidectomy without reimplantation in December 2011 and became largely asymptomatic save for the presence of the tumoral deposits. The parathyroid glands, 4 in total, weighed 0.99 g altogether. Three years later, she had recurrence of tumoral calcinosis, with extensive involvement of the right shoulder (Figure 2), right thigh, and right hip (Figure 3). Despite sodium thiosulphate given at a dose of 25 g thrice weekly via the intraperitoneal route and the cessation of calcium carbonate and alfacalcidol, clinical response was absent. One 30 mg dose of pamidronic acid was then given intravenously to no effect. The huge right shoulder mass, which subsequently became infected and ruptured, revealing chalky calcified material within, was excised with histological confirmation of tumoral calcinosis. She was systemically upset at the time with anorexia, weight loss, and pyrexia. Serum calcium level was also exceedingly high up to 3.88 mol/L. Culture of the postoperative surgical drain fluid yielded *Mycobacterium tuberculosis*. A full course of antituberculosis therapy was then given which resolved her febrile response, but the tumoral calcinosis did not clear. We changed the patient over from CAPD to thrice weekly in-centre hemodialysis (HD) to enhance phosphate clearance, but the tumoral calcinosis worsened over the next year. There was also persistent hypercalcemia (serum Ca 2.73 mmol/L) in the face of low parathyroid hormone levels (iPTH 0.3 pmol/L), normal levels of parathyroid hormone-related protein (PTHrp), and 1,25-hydroxyvitamin D and the absence of paraproteinemia (Table 1). Finally, in view of the likely presence of adynamic bone disease related to antecedent total parathyroidectomy and poor response to the aforementioned efforts to lower calcium and phosphate, teriparatide was given at a dose of 20 micrograms subcutaneously every other day starting January 2017. Since the commencement of teriparatide, there had been progressive reduction in size of the tumoral deposits over the following 2 years, and the patient remained asymptomatic (Figures 4–6). Asymptomatic hypocalcemia with serum calcium dropping to 1.62 mmol/L developed (Table 1) but responded well to the use of oral calcium carbonate and a reduction of teriparatide to 20 micrograms thrice weekly with normalization of serum calcium without ongoing need for calcium carbonate supplementation 2 months later.

## 3. Discussion

We presented a case of refractory tumoral calcinosis developing in a PD patient. The case was difficult to tackle as we had exhausted all conventional therapeutic measures aimed at restoring calcium and phosphate balance. Parathyroidectomy was performed to address worsening SHPT which led to only transient and partial respite. While hypercalcemia and the local inflammation in association with superimposed tuberculous infection of the shoulder lesions might have aggravated the progression of calcified deposits during the first relapse after total parathyroidectomy, the persistent and otherwise unexplained hypercalcemia after the treatment of tuberculous infection was most likely due to the presence of adynamic bone disease, given the low PTH and normal serum 1,25-hydroxyvitamin D levels. Even though a bone biopsy was not performed, we endeavored to exclude secondary causes of hypercalcemia such as multiple myeloma and malignancy. In retrospect, bone metabolism markers and measurement of bone density would have been informative of status of bone turnover, but these were not available at our centre. Our decision to embark on teriparatide therapy was based on a recent report of its use in the treatment of hypercalcemia associated with ABD [12]. There was clinically significant regression of tumoral calcinosis after teriparatide therapy. Severe but asymptomatic hypocalcemia persisted for about 2 months into therapy but responded to oral calcium carbonate. We hypothesize that the intermittent administration of teriparatide increased bone turnover and formation and allowed extraosseous calcium to redistribute back into the skeleton, causing transient hypocalcemia before a new equilibrium occurred. This case report also raises the question of whether parathyroid tissue should be routinely reimplanted at the time of total parathyroidectomy, a procedure that is variably performed across centers.

In conclusion, teriparatide appears to be useful for treating tumoral calcinosis in the presence of adynamic bone disease. Hypocalcemia can occur in the initial months of therapy. More studies are needed regarding the effectiveness and safety of this drug in treating tumoral calcinosis.

## Figures and Tables

**Table 1:** Medications and biochemical values at different time points.

	At diagnosis	Before parathyroidectomy	After parathyroidectomy	Recurrence of tumoral calcinosis	Persistent hypercalcemia	After teriparatide therapy
iPTH (pmol/L)	27.3	64.9	0.3	0.3	0.3	0.3
Calcium (mmol/L)	2.46	2.66	2.44	3.88	2.73	1.62
Phosphate (mmol/L)	4.0	2.8	2.3	2.0	1.8	2.2
ALP (IU/L)	92	103	91	144	152	52
Weekly KT/V from CAPD	2.0	2.4	2.4	2.4	N/A	N/A

iPTH, intact parathyroid hormone; ALP, alkaline phosphatase; CAPD, continuous ambulatory peritoneal dialysis. Calcium is corrected for serum albumin level.

## Data Availability

Underlying data supporting the results of our study can be found by directly emailing the corresponding author of this article.
